# Exosomes induce endolysosomal permeabilization as a gateway by which exosomal tau seeds escape into the cytosol

**DOI:** 10.1007/s00401-020-02254-3

**Published:** 2021-01-08

**Authors:** Juan Carlos Polanco, Gabriel Rhys Hand, Adam Briner, Chuanzhou Li, Jürgen Götz

**Affiliations:** 1 Clem Jones Centre for Ageing Dementia Research (CJCADR), Queensland Brain Institute (QBI), The University of Queensland, Brisbane, QLD 4072 Australia; 2 Present Address: Department of Medical Genetics, School of Basic Medicine and Tongji Medical College, Huazhong University of Science and Technology, Wuhan, 430030 China

**Keywords:** Alzheimer’s disease, Microtubule-associated protein tau, Exosome, Endosome, Lysosome, Spreading, seeding, Autophagy, Protein aggregation

## Abstract

The microtubule-associated protein tau has a critical role in Alzheimer’s disease and other tauopathies. A proposed pathomechanism in the progression of tauopathies is the trans-synaptic spreading of tau seeds, with a role for exosomes which are secretory nanovesicles generated by late endosomes. Our previous work demonstrated that brain-derived exosomes isolated from tau transgenic rTg4510 mice encapsulate tau seeds with the ability to induce tau aggregation in recipient cells. We had also shown that exosomes can hijack the endosomal pathway to spread through interconnected neurons. Here, we reveal how tau seeds contained within internalized exosomes exploit mechanisms of lysosomal degradation to escape the endosome and induce tau aggregation in the cytosol of HEK293T-derived ‘tau biosensor cells’. We found that the majority of the exosome-containing endosomes fused with lysosomes to form endolysosomes. Exosomes induced their permeabilization, irrespective of the presence of tau seeds, or whether the exosomal preparations originated from mouse brains or HEK293T cells. We also found that permeabilization is a conserved mechanism, operating in both non-neuronal tau biosensor cells and primary neurons. However, permeabilization of endolysosomes only occurred in a small fraction of cells, which supports the notion that permeabilization occurs by a thresholded mechanism. Interestingly, tau aggregation was only induced in cells that exhibited permeabilization, presenting this as an escape route of exosomal tau seeds into the cytosol. Overexpression of RAB7, which is required for the formation of endolysosomes, strongly increased tau aggregation. Conversely, inhibition of lysosomal function with alkalinizing agents, or by knocking-down RAB7, decreased tau aggregation. Together, we conclude that the enzymatic activities of lysosomes permeabilize exosomal and endosomal membranes, thereby facilitating access of exosomal tau seeds to cytosolic tau to induce its aggregation. Our data underscore the importance of endosomal membrane integrity in mechanisms of cellular invasion by misfolded proteins that are resistant to lysosomal degradation.

**Supplementary Information:**

The online version contains supplementary material available at 10.1007/s00401-020-02254-3.

## Introduction

Neurodegenerative diseases including Alzheimer’s disease (AD), Parkinson’s disease (PD), Huntington’s disease (HD), frontotemporal lobar degeneration with tau (FTLD-tau) and amyotrophic lateral sclerosis (ALS) are proteinopathies, characterized by the misfolding and aggregation of signature proteins [58]. AD is the most common form of aging dementia, in which extracellular amyloid plaques are formed from fibrillar amyloid-β peptides; whereas, the microtubule-associated protein tau forms intraneuronal fibrillar deposits known as neurofibrillary tangles [15, 58]. An interesting feature of AD is that both pathologies, amyloid-β [70], and in particular Tau [11, 13], occur in patients through well-defined stereotyped stages suggesting spreading. For tau, its pattern of spreading led to the proposition that AD progression may occur by neuron-to-neuron transmission via trans-synaptic transport of misfolded tau seeds from affected to anatomically connected neurons [11, 12]. It is generally believed that a prion-like mechanism is adopted, meaning that misfolded tau seeds actively corrupt the proper folding of soluble tau in recipient cells [51].

Trans-neuronal transfer of tau seeds can be achieved by several mechanisms that involve extracellular vesicles such as exosomes or microvesicles [21, 60, 77], tunneling nanotubes that establish a direct connection between the cytoplasm of neighboring cells [69], or trans-synaptic transfer of membrane-free tau seeds between interconnected neurons [16, 22].

Most of our understanding to date of tau seeding has been obtained using membrane-free tau seeds. However, it is now well established that such seeds are also encapsulated within membranes of exosomes [4, 60, 77], small extracellular vesicles with a diameter of 30–150 nm that are derived from late endosomes known as multivesicular bodies (MVBs) [10, 35]. MVBs are generated by the progressive pinching-off of the endosomal limiting membrane to generate intraluminal nanovesicles that are subsequently stored in the lumen of endosomes. These MVBs which are loaded with intraluminal nanovesicles can fuse with the plasma membrane to release the nanovesicles into the extracellular environment as ‘exosomes’ [10, 35]. Exosomes are systemic messengers that can deliver their cargoes over varying distances, with important physiological roles in the maintenance of cellular homeostasis [67], regulation of an immune response, and even as key mediators of developmental signaling [49, 83, 84]. In a pathological context, exosomes have been mainly studied in cancer, as they regulate the immune response against cancer cells, and promote metastasis [10, 35]. However, growing evidence suggests that exosomes may also be involved in the induction and spreading of pathology in a range of neurodegenerative diseases [35, 82].

We have shown previously that tau biosensor cells, designed to fluorescently display the extent of tau aggregation induced by tau seeds, internalize brain-derived exosomes from rTg4510 tau transgenic mice, forming cytoplasmic puncta with a size reminiscent of endosomes, with tau aggregation induced close to these endosomes and in a threshold-dependent manner [5, 60]. More recently, we also provided evidence that exogenous exosomes are invasive, and that upon their fusion with endogenous endosomes, exosomes can hijack secretory endosomes. In doing so, exosomes can achieve a longer distance of action and a potentially higher pathogenicity in the course of tau spreading [59]. However, a critical, unanswered aspect of this cell-to-cell propagation is how tau seeds contained within an exosome are able to not only exit the exosomal membranes but also escape the endosome to access cytosolic tau and induce corrupting cycles of tau aggregation. Here, we reveal an underlying mechanism by which tau seeds use endosomal permeabilization through lysosomes to interact with cytosolic tau.

## Materials and methods

### Mouse strains and collection of brain tissue

Transgenic rTg4510 mice expressing human four-repeat tau with the P301L mutation linked to hereditary tauopathy [62] and gender-matched wild-type littermate controls were used at 4–6 months of age for exosome isolation from dissected brains. C57BL/6 mice were used at embryonic day 17 (E17) to isolate hippocampal neurons. Animal experimentation was approved by the Animal Ethics Committee of the University of Queensland (approval numbers QBI/412/14/NHMRC and QBI/554/17/NHMRC).

### Plasmids and siRNAs

Plasmids pmCherry-Gal3 (Addgene #85662), pDsRed-Rab7 (Addgene #12661), pLenti6.3/TO/CD9-mEmerald (Addgene #104402), pLV-eGFP (Addgene #36083) and pC0049-EF1a (Addgene # 103865) were a kind gift from Drs. Hemmo Meyer, Richard Pagano, Jens Gruber, Pantelis Tsoulfas and Feng Zhang to Addgene. To generate lentiviral pLV-mCherry-Gal3 driven by the CMV promoter, we directly subcloned the mCherry-Gal3 CDS from plasmid #85662 into plasmid #36083 using the *AgeI* and *SalI* restriction sites. To generate lentiviral pC0049-EF1a-mCherry-Gal3 driven by the EF-1α promoter, we amplified mCherry-Gal3 from plasmid #85662 to introduce *BsiWI* and *EcoRI* restriction sites in the oligos and then cloned the amplification product into plasmid #103865.

For the siRNA-mediated knockdown of RAB7, we used Dharmacon’s siRNA transfection reagents and the pre-designed ON-TARGETplus SMARTpool for human RAB7 (DHA-L-010388-00-0010, Dharmacon) as well as a non-silencing negative control (DHA-D-001810-10-05, Dharmacon). The siRNAs were transfected at a final concentration of 50 nM for 24 h, then after washing and adding the fresh medium, exosomes were delivered dropwise to the cells, further incubated for 24 h when using lipofectamine-mediated uptake, or for 72 h without lipofectamine prior to analysis.

### Isolation and purification of brain exosomes

Exosomes were isolated from the interstitial space of the mouse brain using a previously established protocol [56, 59, 60]. In brief, each brain was chopped and the cells dissociated for 30 min at 37 °C with 0.2% collagenase type III (LS004182, Worthington) in Hibernate-A medium (A1247501, Thermo-Fisher), followed by gentle pipetting with a 10 ml pipette. A series of differential 4 °C centrifugations at 300*g* for 10 min, 2000*g* for 10 min, and 10,000*g* for 30 min was then performed to discard the pellets containing cells, membranes, and nanodebris, respectively. The supernatant from the 10,000*g* centrifugation step was passed through a 0.22-µm syringe filter (Millex-GP, Millipore) and ultracentrifuged at 120,000*g* for 70 min at 4 °C to pellet the exosomes. Pellets from five mouse brains per genotype were pooled, washed with phosphate-buffered saline (PBS, 17-516Q, Lonza) and ultracentrifuged. This preparation of exosome pellets was resuspended in 2 ml of 0.95-M sucrose in 20-mM HEPES (15630-080, Life Technologies), after which a sucrose step gradient (six 2-ml steps: 2.0, 1.65, 1.3, 0.95, 0.6, and 0.25 M on top) was used to purify the exosomes by centrifugation at 200,000*g* for 16 h at 4 °C. Finally, the sucrose-purified exosomes floating in the interphase at 0.95-M sucrose were recovered, washed with 5-ml PBS, ultracentrifuged again, and the exosome pellet resuspended in 120-µl PBS containing 1 × Complete protease inhibitor cocktail (Roche). Protein content was quantified with a BCA™ Protein Assay Kit (23227, Thermo-Fisher) using a 15-µl aliquot of exosomes in PBS, which was mixed with 15 µl of 1 × RIPA buffer (150-mM NaCl, 50-mM Tris–HCl pH7.4, 0.5% (w/v) sodium deoxycholate, 1.0% (v/v) Nonidet P-40, 0.1% (w/v) SDS, 5-mM EDTA, 50-mM NaF) supplemented with protease inhibitors, and then homogenized in a water bath sonicator for 10 min.

### Fluorescent labeling of membranes from brain-derived exosomes

To track exosomes isolated from mouse brains, we labeled their membranes with a fluorescent dye that stably incorporates into the exosome membrane. In our study, we used the fluorescent membrane probes CellVue^®^ Claret Far-Red Fluorescent Membrane Linker (Sigma), PKH67 Green Fluorescent Membrane Linker (Sigma), or CellBrite™ Blue Cytoplasmic Membrane Labeling kit (Biotium) to separately label sucrose-purified exosomes with 0.5–1.0 µl of fluorescent dye in a 500-µl labeling reaction following the manufacturer’s instructions. PD MiniTrap G-25 columns (GE Healthcare Life Sciences) were used to remove potentially unincorporated dye according to the manufacturer’s spin protocol. Finally, labeled exosomes were washed with 6 ml of PBS and concentrated by ultra-centrifugation at 120,000*g* for 70 min, the exosome pellet was resuspended in 120 µl of PBS containing 1 × Complete protease inhibitor cocktail (Roche) and stored at − 20 °C until further use. Protein content of fluorescently labeled exosomes was determined by BCA™ Protein Assay as described above.

### Culture of tau biosensor cells and induction of tau aggregation with exosomes

The ‘tau biosensor cell line’ was kindly provided by Dr. Marc Diamond [34]. This modified monoclonal HEK293T cell line stably expresses two fluorescently tagged forms of the microtubule-binding domain of tau, RD-CFP and RD-YFP [34], thereby allowing for the quantification of tau aggregation by fluorescence resonance energy transfer (FRET) between the two fusion tau proteins, and visualization of tau aggregation detecting tau RD-YFP by confocal microscopy [34, 60]. Cells were grown in DMEM (Dulbecco’s modified Eagle’s medium, 11965092 Thermo-Fisher) supplemented with 10% fetal bovine serum (FBS, SFBS-FR Scientifix), 100 units/ml of penicillin (Thermo-Fisher), 100 µg/ml of streptomycin (Thermo-Fisher), and 2-mM GlutaMAX (Thermo-Fisher). For 24-h assays, cells were seeded at 2 × 10^5^ cells per well in 12-well plates (Corning) overnight, washed and fresh medium was added before treatments with 10-µg protein equivalents of exosomes, prepared in 200-µl Opti-MEM (Thermo-Fisher) containing 5-µl Lipofectamine 2000 (Life Technologies) as previously described [60]. For 72-h assays without lipofectamine, the same number of cells was plated into 6-well plates to avoid over-confluency at 72 h, then treated with 10-µg protein equivalents of exosomes diluted in 500 µl of DMEM culture medium added dropwise.

### Isolation and purification of HEK293T-derived exosomes

Exosomes were isolated from the cell-conditioned medium (CCM) of HEK293T cells cultured in 6X T175 flasks using DMEM supplemented with 5% exosome-depleted fetal bovine serum (edFBS) with supplements as above. The edFBS was prepared by centrifugation of FBS at 120,000*g* for 18 h, followed by filter sterilization of the supernatant. The first collection of CCM was performed when HEK293T cells reached confluency, which was followed by an exchange with a fresh medium containing 1% edFBS. Two additional CCM collections were performed after 24 and 48 h. Each CCM collection was centrifuged at 2000*g* for 10 min, after which the supernatant was centrifuged at 10,000*g* for 30 min. This final supernatant was ultracentrifuged at 120,000*g* for 70 min to pellet exosomes plus potential contaminating proteins. Exosome pellets from three CCM collections were pooled, washed with 25-ml PBS and again ultracentrifuged. The resulting pooled exosome pellet was resuspended in 2 ml of 0.95-M sucrose in 20-mM HEPES (15630-080, Thermo-Fisher), and then purified on a sucrose step gradient column as described above for mouse brain-derived exosomes. Protein content of exosomes was determined by BCA™ Protein Assay as described above.

### FRET flow cytometry

Tau aggregation between RD-CFP and RD-YFP was visualized and quantified by FRET flow cytometry as previously described [34, 60]. In brief, the cells were harvested with 0.05% trypsin–EDTA (Thermo-Fisher), and when required, 40% of dissociated cells were taken out for Western blotting. Dissociated cells intended for flow cytometry were then post-fixed in 2% paraformaldehyde (PFA, Sigma) for 10 min. They were then washed with PBS, pelleted, and resuspended in 1 × Hank’s Balanced Salt Solution (14175095, Thermo-Fisher) containing 1-mM EDTA. We used a FACSAria cell sorter (Becton Dickinson) for FRET analysis, where cells were excited by a 405-nm laser (Coherent Inc.) and the emitted fluorescence was captured with filters for 485/22 nm to detect CFP and 530/30 nm to detect FRET. For each experiment, a total of 40,000 cells were analyzed per replicate using a gating strategy as outlined previously [34, 60]. FRET data were quantified as the integrated FRET signal, calculated by multiplying the percentage of FRET-positive cells in the sample by their respective mean 530-nm fluorescence intensity generated by FRET. For experiments in which RAB7 was labeled with RFP, tau biosensor cells exhibiting red fluorescence were first identified with a PE-Texas Red filter, after which the FRET signal was quantified for the gated red cells only.

### Primary neuronal culture, lentiviral transductions, and exosome treatments

Hippocampal neurons were established from C57BL/6 mice at E17 and grown on 18-mm coverslips coated with poly-D-lysine (PDL) placed in 12-well plates (Corning) [32]. Neurons were plated at a density of 60,000 neurons per well for imaging or 300,000 neurons for western blots, using Neurobasal medium (21103049, Thermo-Fisher) supplemented with 5% serum (FBS; Hyclone), 2% B27 (17504044, Thermo-Fisher), 1 mM GlutaMAX (35050061, Thermo-Fisher), and 50-U/ml penicillin/streptomycin (15070063, Thermo-Fisher). The medium was changed to serum-free Neurobasal medium minus phenol red (12348017, Thermo-Fisher), supplemented with 28-nM 2-mercaptoethanol (21985023, Thermo-Fisher), and 25-µM glutamic acid 24 h post-seeding, and half of the medium was changed twice a week [8, 80]. All cultures were maintained at 37 °C and 5% CO_2_ for up to 10 days in vitro (DIV10). For some experiments, neurons were transduced with lentivirus at DIV2, then treated with exosomes at DIV7 by taking out 500 µl of the neuron-conditioned medium and replacing it with 500-µl fresh Neurobasal culture medium containing 10-µg protein equivalents of exosomes for neurons to be imaged or 20 µg for neurons to be analyzed by western blotting, added dropwise and in the absence of lipofectamine.

### Western blot analysis

Dissociated tau biosensor cells intended for Western blotting were taken out during the preparation of samples for FRET flow cytometry and centrifuged at 1000*g* for 5 min. Then, pelleted cells were sonicated in RIPA buffer (150-mM NaCl, 50-mM Tris–HCl pH7.4, 0.5% (w/v) sodium deoxycholate, 1.0% (v/v) Nonidet P-40, 0.1% (w/v) SDS, 5-mM EDTA, 50-mM NaF) supplemented with 1 × complete protease inhibitors (Roche). A similar procedure was performed with hippocampal primary neurons [8]. Protein content was quantified with a BCA™ Protein Assay Kit (23227, Thermo-Fisher), to separate 10–20 µg of protein by 7–12% SDS-PAGE electrophoresis, which was then transferred onto Immuno-Blot low fluorescence PVDF membranes (170-4275, Bio-Rad) using the Trans-Blot Turbo transfer system (Bio-Rad). Membranes were blocked in Odyssey Blocking Buffer (Li-Cor) for 1 h at room temperature (RT) and then incubated overnight at 4 °C with primary antibodies prepared in a 1:1 mixture of Odyssey Blocking Buffer and Tris-buffered saline/0.1% Tween-20 (TBST). Membranes were washed with TBST three times for 10 min at RT, followed by a 1-h incubation with IRDye secondary antibodies (Li-Cor) diluted 1:10,000 in the same buffer as used for the primary antibodies. Finally, membranes were again washed three times in TBST and the fluorescence signals were recorded using an Odyssey Fc imaging system (Li-Cor). Analysis and protein quantification was performed using Image Studio software (Li-Cor). The following antibodies were used: Rab7 (D95F2) XP-rabbit antibody (1:1000; #9367, CST), anti-LC3B Rabbit antibody (1:1000; 2775 Cell Signaling) and the normalizer anti-GAPDH mouse antibody (1:3000; MAB374; Millipore).

### Production of active lentiviral particles

Lentiviral constructs were used to generate active viral particles by transfecting Lenti-X cells (632180, Takara Bio) with third-generation lentiviral packaging system plasmids [23]. Transfection of plasmids was performed either by CaPO_4_ precipitation [73] or with TransIT-VirusGEN (MIR6700, Mirus) according to the manufacturer’s instructions. Transfection mixture was added to adherent Lenti-X cells cultured in DMEM with pyruvate (11995073, Thermo-Fisher) containing 10% FBS (Scientifix), the lentivirus-containing medium was collected after 48 h, centrifuged at 3000*g*, filtered at 0.45 µm and centrifuged again 10,000*g* for 4 h at 4 °C. For this final centrifugation, the lentivirus-containing medium was suspended above a 10% sucrose cushion (100-mM NaCl, 0.5-mM EDTA, 10% sucrose, 50-mM Tris–HCl to pH 7.4). The supernatant was discarded, the lentiviral pellet was resuspended in 160 µl of 1 × HBSS (Hank’s Balanced Salt Solution, 14175095 Thermo-Fisher) and 20-µl aliquots were snap-frozen in liquid nitrogen and then stored at − 80 °C. Lentiviral titer was then calculated by treating adherent HEK293T cells with serial dilutions of thawed lentivirus. These cells were dissociated 3 days post-transduction and resuspended in 1 × HBSS containing 1-mM EDTA. Fluorescent cells were quantified with flow cytometry using a BD LSR Cell Analyzer (Becton Dickinson), and transducing units per milliliter were calculated based on relative fluorescence events [73].

### Generation of stable lentiviral cell lines

To generate cells stably expressing pLenti6.3/TO/CD9-mEmerald, HEK293T cells were cultured in DMEM with 10% FBS for 24 h, transduced with active lentivirus at an MOI of 20, and cultured for a further 24 h after which the media were replaced with fresh DMEM containing 10% FBS. After another 24 h, the cells were passaged and selected by culturing in DMEM containing 10% FBS and 5-µg/ml blasticidin for 72 h, then passaged and sorted by fluorescence with flow cytometry using a BD Influx™ Cell Sorter (BD Biosciences). To generate stable pLV-mCherry-Gal3-expressing cells (HEK293T and tau biosensor cells), the same procedure was followed excluding blasticidin selection, as the pLV-mCherry-Gal3 plasmid does not confer blasticidin resistance. The selection was repeated until ≥ 99% of cells showed above-threshold fluorescence during flow cytometry.

### Immunofluorescence of cells on coverslips

Cells were grown on 18-mm coverslips with a PDL coating, in a DMEM culture media with 2% edFBS, then treated with fluorescently labeled exosomes as described above. After exosome internalization, cells were washed 3 times with PBS, fixed with 1 ml of 4% paraformaldehyde in PBS for 20 min at RT, and then washed with PBS containing 200-mM glycine. Fixed cells were permeabilized for 30 min with PBS containing 0.2% saponin and 3% bovine serum albumin (BSA). Primary and secondary antibodies were diluted in a solution of PBS containing 0.1% saponin and 1% BSA. Three washes post antibodies were performed with PBS with 0.1% saponin. Primary antibodies were anti-LC3A/B Rabbit mAb (1:100; 12741 Cell Signaling), anti-LC3B Rabbit antibody (1:200; 2775 Cell Signaling). Secondary antibody was Alexa Fluor 647 goat anti-rabbit (1:1000, A21245 Thermo-Fisher). Coverslips were mounted on Superfrost Plus slides (Menzel-Glaser) using VectaShield Antifade mounting medium (H1000 Vector).

### Detection of lysosomes and alkalinization

We used the marker LAMP1 (lysosomal-associated membrane protein 1) fused with RFP to detect lysosomes and endolysosomes, transducing either neurons or HEK293T cells with a baculoviral vector (C10597, Thermo-Fisher) for 48 h followed by confocal microscopy analysis of fixed cells. To track acidic lysosomes, cells were treated with LysoTracker^®^ Deep Red (L12492, Thermo-Fisher) at 66 nM concentration in the culture medium, and incubated at 5% CO_2_ and 37 °C for 1 h. The cells were then washed 3 times with PBS prior to fixation and confocal imaging. To test the effect of alkalinization on lysosomes, the tau biosensor cells were treated with exosomes in culture media containing ammonium chloride (50 mM).

### Confocal microscopy and image analysis

Image acquisitions were performed with a 63X objective using a Zeiss LSM 710 Inverted Laser Scanning Confocal Microscope, or with a 100X objective and 2 × optical zoom using a spinning disk confocal microscope (Diskovery; Andor Technology) built around a Nikon Ti-E body (Nikon Corporation). Images from the spinning disk were subsequently deconvoluted with Huygens Professional (Scientific Volume Imaging) to increase lateral resolution and dynamic range for more accurate 3D colocalization. Deconvoluted images were imported into Imaris v9.5.1 (Bitplane), in which 3D masking was applied to cells to exclude signal outside of the cytoplasm and within the nucleus, leaving only cytoplasmic signal for colocalization and puncta quantifications.

For tau biosensor cells, fluorescence colocalization analysis was carried out with Imaris software v9.5.1 (Bitplane) using a thresholding algorithm to calculate the Manders’ colocalization coefficients representing the fraction of each fluorophore colocalizing with the other fluorophore (M1 and M2). For neurons, 32 bit 3D masked images were then colocalized with bisection automatic thresholding ignoring zero value pixels (masked pixels) using the Coloc 2 plugin in ImageJ 1.53c to generate the Manders’ coefficients M1 and M2. Imaris software was also used for the quantification of fluorescent puncta representing endosomes or lysosomes, using non-overlapping confocal images in which the acquisition parameters remained invariable for all the images showing the internalization untreated control compared with cells treated with exosomes. A representative image containing fluorescent puncta in two channels (i.e., wild-type exosomal endosomes in the green channel and lysosomes in the red channel) was used to create surfaces specific for each fluorescence channel and specific image segmentation algorithms to detect and quantify fluorescent puncta in each channel. The resulting segmentation algorithm was then applied to all other images. Colocalization analysis was always performed at an individual cell level, and when more than one cell was present in an image (i.e., HEK293T cells), an individual contour adjusted to individual cells was drawn to create a surface representing a region of interest (ROI), which was then used to mask each channel and run segmentation algorithms inside that specific ROI only.

Quantification of LC3 fluorescence intensity in primary neurons was carried out with ImageJ on single optical sections captured on the Zeiss LSM 710 confocal microscope described above. Representative perinuclear ROIs ending at the somatic border were selected to exclude nuclei from analysis, then mean gray value and the area (as a percentage of the ROI) occupied by signal above threshold was measured. A consistent threshold was determined in ImageJ by automatic Yen thresholding in several experimental images, then the same threshold was applied to all images analyzed. Corrected mean fluorescence was calculated by measuring and subtracting the mean gray value of another ROI containing only background signal from the cellular mean fluorescence. Similarly, LC3 whole-cell signal quantifications for HEK293T cells were performed using ImageJ software as previously described [29].

### Statistical analysis

To determine the statistical significance of differences in quantification levels, *p*-values either were determined from a two-tailed unpaired *t*-test with Welch’s correction or from one-way ANOVA analysis with a 95% confidence interval and Dunnett’s or Tukey’s test to correct for multiple comparisons, calculated with GraphPad Prism v8.3 for Windows (GraphPad Software Inc).

## Results

### Endosomes containing brain-derived exosomes mostly fuse with lysosomes

Amyloid assemblies of tau and α-synuclein (a hallmark pathology of PD) share the ability to rupture endocytic vesicles after internalization, and both are targeted to lysosomes [27]. Lysosomal activity has been shown in cellular models to be critical for the ability of α-synuclein fibrils to seed intracellular aggregates [74]. Given that, similar to these membrane-less protein aggregates [27], exosomes are also endocytosed and mostly fuse with lysosomes [24, 59, 72], we hypothesized that lysosomes may facilitate the escape of exosomal tau seeds from endosomes.

When lysosomes fuse with endosomes, a hybrid organelle is formed known as endolysosome [46, 79]. We first addressed whether lysosomes are colocalized with internalized exogenous exosomes which had been isolated from P301L tau transgenic rTg4510 and wild-type brains [59, 60]. For that, we used a widely used non-neuronal cellular model known as tau biosensor cells [34, 60], as well as primary mouse neurons [59].

**Fig. 1:**
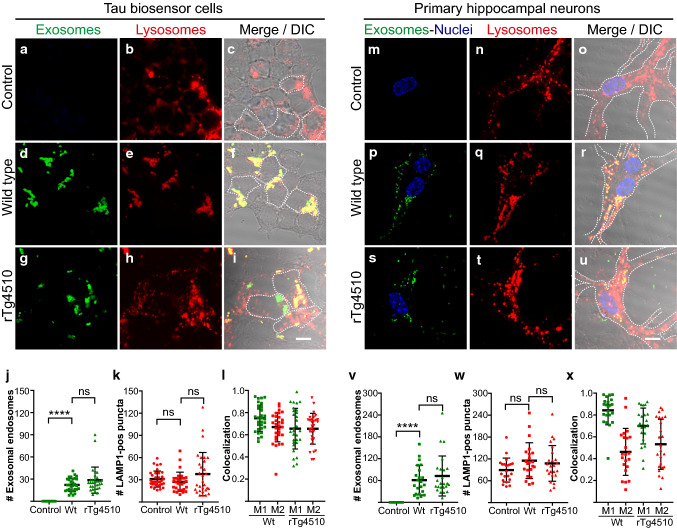
The majority of endosomes containing brain-derived exosomes fuse with lysosomes. Tau biosensor cells and hippocampal neurons expressing a lysosomal marker (RFP-tagged LAMP1) were treated with brain-derived exosomes, which had been labeled with two fluorescent membrane dyes, either CellBrite™ blue (pseudo-colored green for better visualization) or PKH67 (green). Confocal images were taken 24 h post-treatment. Selected individual cells are outlined with dashed lines. **a**–**c** Control tau biosensor cells without exosome treatment display the physiological distribution of lysosomes labeled with LAMP1-RFP. **d**–**f** Tau biosensor cells treated with exosomes from wild-type brains reveal colocalization with the lysosomal marker. **g**–**i** rTg4510-derived exosomes also colocalize with lysosomes. **j** Quantification of numbers of exosome-containing endosomes per cell showing similar uptake for wild-type and rTg4510 exosomes. **k** LAMP1-positive subcellular compartments or puncta per cell remained statistically similar between untreated control cells and cells internalizing exosomes. Error bars represent ± SD for 30 individual cells analyzed from four independent experiments. ****p < 0.0001; ns, not significant. **l** Manders’ colocalization coefficients, with M1 representing the fraction of the exosomal signal (green) that colocalizes with the lysosomal LAMP1 signal (red), and M2 the fraction of the lysosomal signal that colocalizes with the exosomal signal. **m**–**o** Control neurons showing LAMP1-expressing lysosomes. **p**–**x** Colocalization of exosomes and lysosomes is also evident in primary hippocampal neurons treated with exosomes derived from wild-type (**p**–**r**) or rTg4510 brains (**s**–**u**). Quantification of exosome-containing endosomes per cell (**v**) and LAMP1-positive subcellular compartments or puncta per cell (**w**) in hippocampal neurons. Error bars represent ± SD for 25 individual neurons analyzed from four independent experiments. ****p < 0.0001; ns, not significant. **x** Manders’ colocalization coefficients as above. Scale bar: 10 µm for all panels

Following transduction of tau biosensor cells with a baculovirus for expression of the lysosomal marker protein LAMP1 (lysosomal-associated membrane protein 1) tagged with RFP for visualization, we first assessed whether endocytosed exosomes and endogenous lysosomes would colocalize and if so to which extent, by determining Manders’ coefficients. Manders’ M1 determines the proportion of endocytic compartments that contain exosomes and also express LAMP1; whereas, Manders’ M2 determines the cellular fraction of the lysosomal marker LAMP1 that is colocalized with exosomes. We found 24 h after treatment with exosomes (Fig. 1), based on M1 coefficients, that ~ 75% of endosomes containing wild-type exosomes or 65% of endosomes containing rTg4510-derived exosomes exhibited expression of the lysosomal marker LAMP1-RFP, indicating that they were endolysosomes (Fig. 1a–l). Similarly, M2 revealed that on average 66% of lysosomes were found to be colocalized with exosome-containing endosomes (Fig. 1l). In primary hippocampal neurons, M1 coefficients showed that 84% of endosomes containing wild-type exosomes or 70% containing rTg4510-derived exosomes colocalized with LAMP1-RFP (Fig. 1m–x), suggesting that the formation of endolysosomes triggered by internalized exosomes is a conserved cellular mechanism.

### Acidic endolysosomes are required for tau aggregation

**Fig. 2:**
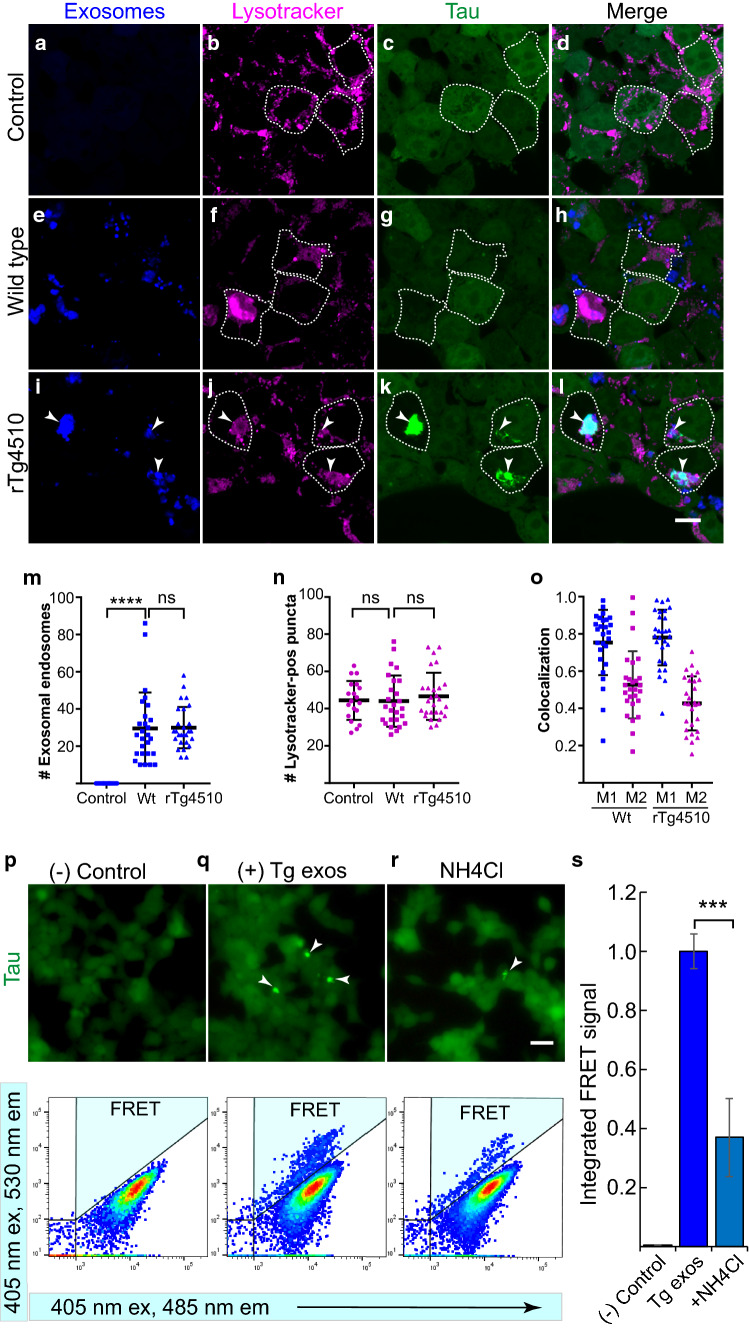
Low pH endolysosomes are generated and required during tau aggregation induced by exosomes. Lysotracker Deep Red (magenta) reveals the low pH of the endocytic organelles containing exosomes (blue, labeled with CellBrite™) in tau biosensor cells displaying tau RD-YFP in green. Scale bar: 10 µm. **a**–**d** Control tau biosensor cells not treated with exosomes contain acidic lysosomes. **e**–**h** 24 h after treatment, endosomes containing wild-type-derived exosomes colocalize with the acidic Lysotracker probe. **i**–**l** Similarly, rTg4510-derived exosomes colocalize with Lysotracker-positive endolysosomes, around which tau aggregates are induced (arrowheads). **m** Quantification of the number of exosome-containing endosomes in individual tau biosensor cells does not differ between wild-type and rTg4510 exosomes. **n** Lysotracker-positive subcellular compartments or puncta per cell are similar for all groups. Error bars represent ± SD for 29 individual cells analyzed from four independent experiments. ****p < 0.0001; ns, not significant. **o** Colocalization, in that M1 represents the proportion of exosomal signal (blue dots) that colocalizes with the Lysotracker signal, and M2 the fraction of the Lysotracker signal (purple dots) colocalizing with the exosomal fluorophore in tau biosensor cells treated with wild-type (Wt) or rTg4510-derived (Tg) exosomes. **p**–**s** Increasing the cellular pH of tau biosensor cells with the alkalinizing agent ammonium chloride (NH_4_Cl) reduces tau aggregation induced by exosomes: (**p**–**r** top panels) Epifluorescence microscopy detecting tau RD-YFP in cells before flow cytometry. Brighter spots (arrowheads), representing tau aggregates in cells treated with rTg4510-derived exosomes, decrease with NH_4_Cl treatment. Scale bar: 50 µm. (**p**–**r** bottom panels) Representative flow cytometry plots (highlighting in blue the quadrant in which the FRET signal caused by tau aggregation was detected and quantified). **p** Control cells showing absence of a FRET signal. **q** Positive control by adding rTg4510-derived exosomes (Tg exos, 10-µg protein equivalent), resulting in a strong FRET signal. **r** Treatment with 50-mM NH_4_Cl massively reduces the FRET signal induced by rTg4510-derived exosomes. **s** Quantification of the integrated FRET signal normalized to the signal detected from the positive control with rTg4510-derived exosomes (Tg exos). Alkalinization with NH_4_Cl produces a strong reduction in tau aggregation measured by FRET. Error bars represent SEM for *n* = 3, 40,000 cells per individual experiment, ***p  < 0.001

Having demonstrated that the majority of endocytic compartments which contain exosomes fuse with lysosomes to form endolysosomes, we set out to analyze whether the latter were acidic, given that the activity of lysosomal enzymes in endolysosomes depends on a low pH of 4.5–6.0 [46, 79]. We determined the acidic status of exosome-containing endosomes using LysoTracker^®^ Deep Red (Fig. 2), a far-red fluorescent dye that is routinely used to label and track acidic organelles such as lysosomes [41]. Manders’ M1 colocalization analysis revealed that 75% of endosomes containing wild-type exosomes, or 78% containing rTg4510-derived exosomes colocalized with the LysoTracker probe (Fig. 2e–o), which supports the notion that exosomes are in acidic endolysosomes where the principal activity of lysosomal enzymes occurs at a low pH [14, 41]. Tau aggregation occurred around these acidic endolysosomes containing rTg4510-derived exosomes (white arrowheads, Fig. 2i–l; Supplementary Fig. 1), indicating that the normally degradative activities of lysosomal enzymes failed to degrade the exosomal tau seeds and prevent the induction of tau aggregation. Moreover, wild-type- and rTg4510-derived exosomes were endocytosed at a similar rate (Fig. 2m), and consequently, the numbers of LysoTracker-positive endolysosomes were also very similar for both treatments (Fig. 2n). Colocalization analysis showed an average of 77% exosome-containing endosomes colocalizing with LysoTracker to form acidic endolysosomes (Fig. 2o). Together, the robust colocalization of brain-derived exosomes with LAMP1-RFP-labeled lysosomes (Fig. 1), and the LysoTracker-positivity of exosome-containing endocytic compartments (Fig. 2), indicate that internalized exosomes are mainly located in fully active endolysosomes which constitute a favorable environment for lysosomal activity.

In tau biosensor cells, tau aggregation can be quantified by fluorescence resonance energy transfer (FRET) between the tau RD-CFP and RD-YFP fusion proteins which these cells express [34, 60]. FRET energy transfer only occurs when two proteins of interest are very close, typically within 2–6 nm, as is the case for tau aggregates. We asked whether tau aggregation would be impaired when lysosomal enzymes were inactivated, which can be achieved by increasing the pH. Using the alkalinizing agent ammonium chloride (Fig. 2p–s), the ability of rTg4510-derived exosomes to induce tau aggregation was measured by FRET [34, 60] and fluorescence microscopy: differences in the profiles of tau aggregates were evident by epifluorescence microscopy of the treated cells immediately before they underwent cell sorting (Fig. 2p–r top panels), as well as by FRET flow cytometry (Fig. 2p–r bottom panels). By measuring FRET intensity and the number of FRET-positive cells, we quantified the level of tau aggregation in a large number of cells as previously described [34, 45]. We found that whereas rTg4510-derived exosomes induced robust tau aggregation, this was strongly reduced (by 63%) in the presence of ammonium chloride (Fig. 2s). This suggests that the low pH required for proper lysosomal activity facilitates the escape of exosomal tau seeds from endolysosomes to induce aggregation.

### Gain- and loss-of-function of RAB7 further supports the role of lysosomes in tau aggregation

**Fig. 3:**
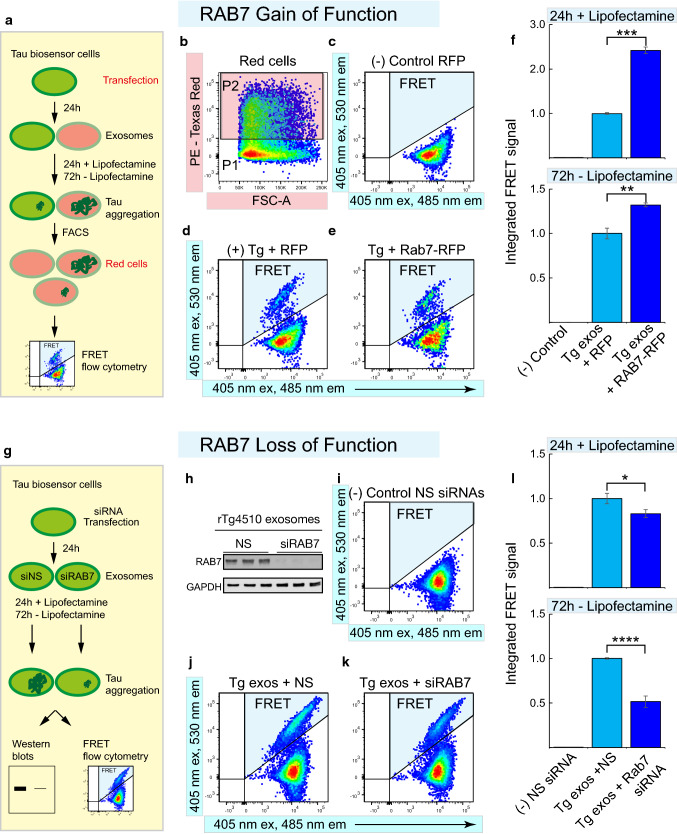
Overexpression and knockdown of RAB7 support a role for lysosomes in tau aggregation induced by exosomal tau. **a** Diagram of gain-of-function assays using RAB7-RFP overexpression, analyzed either after 24 h using lipofectamine-mediated uptake of exosomes or after 72 h without using lipofectamine. **b**–**f** RFP-tagged RAB7 strongly enhances tau aggregation in tau biosensor cells treated with rTg4510-derived exosomes (Tg): **b** Flow cytometry plot showing the identification of RFP-expressing cells (P2, pink top gate), which were then analyzed for FRET between tau RD-CFP and tau RD-YFP. (**c**) Cells expressing only RFP and treated with wild-type exosomes served as a negative control (highlighting in blue the quadrant in which the FRET signal should be detected and quantified). **d** FRET-positive control consisting of cells expressing only RFP and treated with rTg4510-derived exosomes. **e** RAB7-RFP-expressing cells treated with rTg4510-derived exosomes results in a strong increase in FRET-positive cells. **f** Quantification of the integrated FRET signal normalized to the signal detected for Tg exos + RFP. Overexpression of RAB7 (with or without lipofectamine) resulted in a strong increase in tau aggregation measured by FRET. Error bars represent SEM for *n* = 3, 40,000 cells per individual experiment, ***p < 0.001; **p  < 0.01. **g** Experimental design for RAB7 loss-of-function assays. **h**–**l** Knockdown of endogenous RAB7 in tau biosensor cells using a pool of commercially available RAB7-specific siRNAs results is a strong reduction of tau aggregation induced by exosomes: (**h**) Western blot analysis of a fraction of the cells used for FRET analysis, corroborating an effective knockdown of RAB7 (NS, non-silencing siRNA pool; siRAB7, the pool of RAB7-specific silencing siRNAs). **i** Wild-type exosomes plus non-silencing siRNAs serving as a negative control, reveal an absence of a FRET signal. **j** rTg4510-derived exosomes (Tg exos) plus non-silencing siRNAs (NS) used as a positive control, yielding a strong FRET signal. **k** Treatment with rTg4510-derived exosomes plus RAB7-specific silencing siRNAs (siRAB7) showing a decrease in FRET cells. **l** Quantification of the integrated FRET signal normalized to the signal detected for Tg exos + NS, showing that RAB7 knockdown results in a significant decrease in tau aggregation, a decrease that is stronger when lipofectamine is not used for the exosomal uptake. Error bars represent SEM for *n* = 3, *p < 0.05; ****p  < 0.0001

It is well established that the GTPase RAB7 is required for the fusion of endosomes with lysosomes to generate endolysosomes [36, 41, 46]. We hypothesized that tau aggregation induced by exosome-containing endosomes would be altered by modulating their fusion with lysosomes. To test this, we manipulated RAB7 by performing both gain- and loss-of-function studies using tau biosensor cells (Fig. 3).

Tau biosensor cells can reveal the differences in seeding potential from samples containing tau seeds under different experimental conditions [34, 60]. Lipofectamine-mediated uptake strongly increases the sensitivity of this system and accelerates the seeding reaction [34, 60]. Typically, 24 h are sufficient to obtain a strong induction of tau aggregation using lipofectamine, whereas 72 h are required when lipofectamine is not used [60]. We used tau biosensor cells expressing either RFP-labeled RAB7 or an RFP-expressing control vector, and treated them with rTg4510-derived exosomes with lipofectamine for 24 h or without lipofectamine for 72 h to control for potential confounds of lipofectamine (Fig. 3a). By sorting RFP-positive cells by flow cytometry, followed by FRET analysis (Fig. 3b), we found that cells expressing only RFP and treated with wild-type exosomes generated no FRET signal, as expected (Fig. 3c). In contrast, rTg4510-derived exosomes induced a clear FRET signal in cells transfected with the RFP-vector control, representing tau aggregation (Fig. 3d). Importantly, tau aggregation was strongly increased when rTg4510-derived exosomes were added to the cells expressing RFP-labeled RAB7 (Fig. 3e, f). These data support the notion that RAB7-mediated fusion with lysosomes indeed increases tau aggregation. It is worth mentioning that the RAB7-mediated increase in tau aggregation was induced irrespective of whether lipofectamine was used, although lipofectamine enhanced the level of tau aggregation (Fig. 3f).

We next performed a loss-of-function analysis of endogenous RAB7 using commercially available RAB7-specific siRNAs (Fig. 3g–l). Tau biosensor cells were treated with the siRNAs for 24 h and exosomes were added for another 24 h with lipofectamine or for 72 h without lipofectamine, after which a FRET flow cytometry analysis was performed (Fig. 3g). To ensure that sufficient time had elapsed for the siRNAs to exert their effect, a fraction of the cells to be analyzed by flow cytometry was recovered for western blot analysis, which confirmed an 85% knockdown in RAB7 protein expression (Fig. 3h). In the negative control containing wild-type exosomes and non-silencing siRNAs, we did not observe a FRET signal (Fig. 3i). Adding the rTg4510-derived exosomes to the non-silenced cells generated a FRET signal (Fig. 3j). However, when RAB7-knockdown cells were treated with rTg4510-derived exosomes, a significant decrease in tau aggregation was observed (Fig. 3k–l). Notably, lipofectamine did not change the negative effect of the RAB7 knockdown on tau aggregation but appeared to enhance tau aggregation even under knockdown conditions (Fig. 3l).These results further indicate that lipofectamine enhances tau aggregation irrespective of the functional assay we have used, potentially by increasing the fluidity of biological membranes as has been previously proposed [6], or by increasing exosomal uptake [60]. Together, the above findings from both gain- and loss-of-function studies support the notion that lysosomal function mediated by RAB7 is important for exosome-induced tau aggregation.

### Recruitment of lysosomes by exosome-containing endosomes results in permeabilization which is used by tau seeds to access the cytosol

Having demonstrated that lysosomes are important for exosome-induced tau aggregation, we next asked how the activity of lysosomes, intended to degrade endocytosed material, might facilitate a process by which exosomal tau seeds are not degraded and instead escape from the endolysosomes. As described above, exosomes appear to persist even after fusion with lysosomes (Fig. 1), and even after the induction of tau aggregation, some labels of exosomal membranes were still detectable (Fig. 2i–l), suggesting integrity of the exosomal membranes. We reasoned that the complete degradation of exosomal membranes, which have a unique lipid composition conferring high resistance to degradation [65, 84], would require a strong and prolonged activity of lysosomal enzymes. In this model, we hypothesized that the lengthy process of the lysosomal activity required to degrade the exosomes contained within the endolysosomes would cause transient permeabilization of the endosomal membranes. This would be similar to the rupture of endolysosomes that is actively triggered by incoming pathogens [71], or that arises incidentally by membrane destabilizing molecules such as crystals [66], or membrane-less protein aggregates characteristic of neurodegenerative diseases [27, 40]. Permeabilized endolysosomes can be detected by cytosolic sensors such as galectins, and the formation of galectin puncta has proven to be a sensitive way to demonstrate lysosome-mediated permeabilization of endocytic organelles [3, 27, 47, 55]. Galectins bind β-galactose-containing glycoconjugates, which are either present on the cell surface or on the luminal side of endosomes [3, 37, 47, 66, 71]. Galectins are normally diffusely distributed throughout the cytoplasm and can only access the lumen of endosomes when these organelles become permeabilized, generating characteristic puncta that colocalize with endosomal and lysosomal markers [3, 47].

**Fig. 4:**
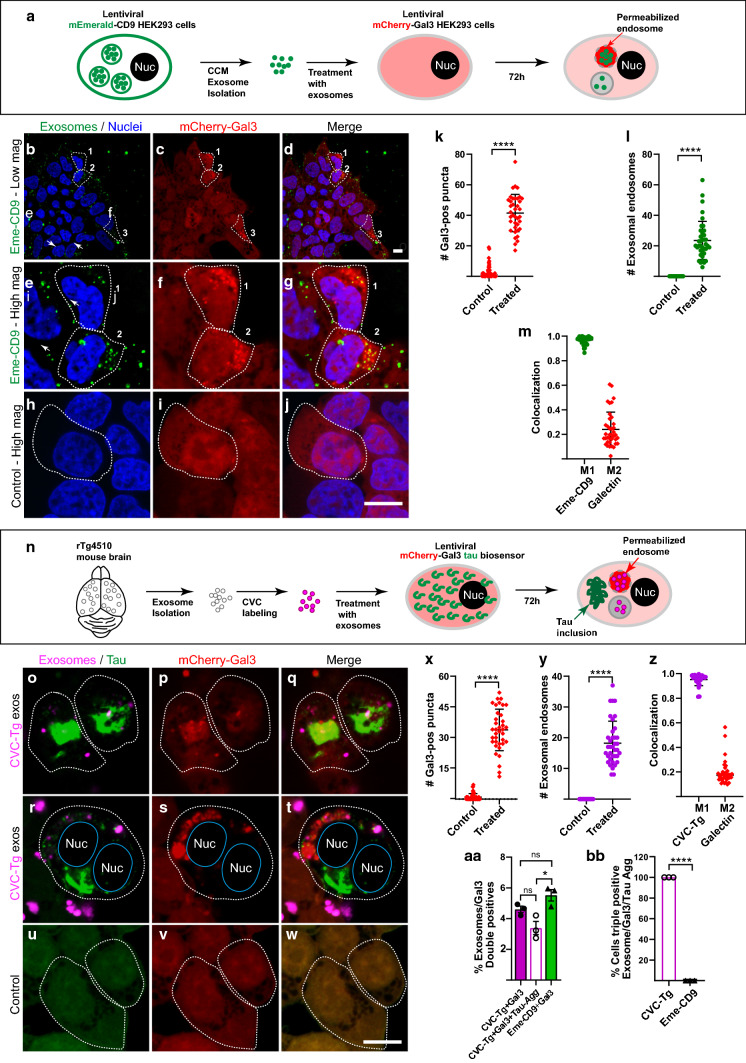
Endosomal membrane permeabilization is triggered by exosomes in HEK293T cellular models. **a** Experimental diagram showing the generation of lentiviral-derived HEK293T cell lines to produce exosomes labeled with mEmerald-tagged CD9 (Eme-CD9), which were used to treat lentiviral HEK293T cells expressing galectin-3 tagged with mCherry (mCherry-Gal3) for 72 h without lipofectamine. **b**–**m** Treatment with Eme-CD9 exosomes results in the permeabilization of endosomes in a small fraction of cells. Selected individual cells are outlined with dashed lines. Scale bar is 10 µm for all panels: **b**–**d** Low magnification image showing galectin puncta, representing permeabilized endosomes, which are only detected in a limited number of cells (dashed lines, indicated 1, 2, 3), although all cells appear to have internalized Eme-CD9 exosomes. **e**–**g** High magnification image showing that cells with galectin puncta contain Eme-CD9 exosomes. **h**–**j** Most untreated control cells do not show galectin puncta. **k** Quantification of the number of galectin puncta per individual cell reveals a strong increase following treatment with Eme-CD9 exosomes. **l** Individual cells showing exosome-containing puncta that are only detected in treated cells. Error bars represent ± SD for 38 individual cells analyzed from three independent experiments. ****p < 0.0001. **m** Manders’ colocalization coefficients, with M1 representing the fraction of the exosomal signal (green circles) that colocalizes with mCherry-Gal3. M2 is the fraction of the mCherry-Gal3 signal (red diamonds) colocalizing with the exosomal green fluorophore. **n** Diagram showing isolation of rTg4510-derived brain exosomes labeled with CellVue claret Far-red dye (CVC-Tg exos) used to treat tau biosensor cells expressing lentiviral mCherry-Gal3. **o**–**q** Treatment with CVC-Tg exosomes results in galectin puncta containing internalized exosomes, which showed the ability to induce tau aggregation in the cytosol (bright green signal of tau-YFP). **r**–**t** Tau biosensor cell apparently dividing as it harbors two nuclei (Nuc) despite the presence of mCherry-Gal3 puncta and induced tau aggregates in the cytosol. **u**–**w** Control cells did not show galectin puncta or tau aggregation. **x** Galectin puncta per individual cell strongly increase with the treatment with CVC-Tg exosomes. **y** Exosome-containing puncta are only detected in cells treated with CVC-Tg exosomes. Error bars represent ± SD for 37 individual cells analyzed from three independent experiments. ****p < 0.0001. **z** Colocalization showing M1 representing the fraction of the exosomal signal (purple dots) that colocalizes with Cherry-Gal3. M2 is the fraction of the mCherry-Gal3 signal (red diamonds) colocalizing with the exosomal far-red fluorophore. **aa** Comparative quantification of the percentage of cells showing both galectin and exosomal puncta (double positives) in both mCherry-Gal3 lentiviral cell lines described in **a** and **n**. Tau biosensor cells (purple column) and HEK293T cells (green column) show a similar percentage of double positives. 76% of tau biosensor cells with double-positive puncta developed tau aggregates, whereas 24% were permeabilized without forming aggregates (white middle column, + Tau-*Agg*). **bb** Quantification of triple positive cells (exosomes + Gal3 + Tau-*Agg*) within the double-positive subpopulations (exosomes + Gal3) described in **aa**. 100% of tau biosensor cells treated with CVC-labeled tau transgenic exosomes were triple positive, supporting that tau aggregation only occurs in the presence of permeabilization. Error bars represent ± SEM for n = 3 of 1,514 cells analyzed. *p  < 0.05; ****p < 0.0001; *ns* not significant

For investigating whether exosome-containing endosomes become permeabilized, we transduced HEK293T and tau biosensor cells with a lentivirus encoding a mCherry-Gal3 fusion protein, which generated polyclonal cell lines. We then treated the cells with two types of exosomes, either (i) tau-free exosomes isolated from conditioned media of a lentiviral-derived HEK293T stable cell line expressing the exosomal marker CD9 labeled with the monomeric Emerald green (Eme-CD9) fluorescent protein located in the luminal side of the vesicles [9]; or (ii) tau-containing exosomes derived from rTg4510 brains and labeled with fluorescent membrane-intercalating dyes [59, 60]. Importantly, these treatments were performed for 72 h without lipofectamine (Fig. 4) to avoid potential confounding effects of endosomal permeabilization caused by a lipofectamine-mediated increase in membrane fluidity [6, 17]. It is worth mentioning that we switched to Eme-CD9 exosomes to replace brain-derived wild-type exosomes as a control for the following reasons: (i) to address whether the potential permeabilization could be triggered by exosomes that do not contain any form of tau, and (ii) to control whether the membrane-intercalating dyes used to label brain-derived exosomes would display differential permeabilization when compared with exosomes labeled with a fluorescent protein (Eme-CD9).

We first treated HEK293T-mCherry-Gal3 cells with HEK293T-derived exosomes labeled with Eme-CD9 (Fig. 4a–g). We observed that all cells had internalized Eme-CD9 exosomes but only a few showed mCherry-Gal3 puncta, indicative of endosomal permeabilization (Fig. 4b–d). High magnification images of the cells exhibiting galectin puncta revealed colocalization with internalized Eme-CD9 exosomes (Fig. 4e–g); whereas, the control cells had no galectin puncta (Fig. 4h–j). Quantification of Gal3 puncta in a number of individual cells showed that in the absence of exogenous exosomes, the majority of control HEK293T-mCherry-Gal3 cells exhibited no Gal3 puncta, although a few cells showed minor numbers of puncta suggesting some level of physiological permeabilization (Fig. 4k). However, after treatment with Eme-CD9 exosomes, many Gal3 puncta were developed (Fig. 4k). As expected, exosome-containing endosomes were only observed in cells that were treated with Eme-CD9 exosomes (Fig. 4l). Furthermore, in the cells showing galectin puncta, the M1 colocalization coefficient indicated that 96% of the endosomes carrying Eme-CD9 exosomes showed colocalization with mCherry-Gal3; whereas, only 24% of the total mCherry-Gal3 signal colocalized within exosome-containing endosomes, with the rest of mCherry-Gal3 remaining in the cytosol (Fig. 4m).

Next, we used tau biosensor cells that we made to endogenously express the lentiviral mCherry-Gal3 fusion protein, and treated them with brain-derived exosomes from rTg4510 mice, labeled with the membrane-intercalating far-red fluorescent dye CellVue Claret (CVC). We asked whether the induction of tau aggregation was linked to the triggering of endolysosomal permeabilization revealed by mCherry-Gal3 puncta (Fig. 4n). We found that CVC-labeled tau transgenic exosomes induced tau aggregation (Fig. 4o–t), which formed around exosome-containing endosomes exhibiting a strong mCherry-Gal3 signal, demonstrating permeabilization of these endolysosomes (Fig. 4o–t). In contrast, untreated mCherry-Gal3 cells neither showed galectin puncta nor tau aggregation (Fig. 4u–w). Tau biosensor cells expressing mCherry-Gal3 exhibited a strong increase in Gal3 puncta after treatment with CVC-labeled tau transgenic exosomes (Fig. 4x). Similar to Eme-CD9 exosomes, the CVC-labeled tau transgenic exosomes in tau biosensor cells showing galectin puncta exhibited a strong M1 colocalization coefficient of 95%; whereas, only 19% of the total mCherry-Gal3 signal colocalized within exosome-containing endosomes (Fig. 4z). We also quantified the percentage of cells with galectin puncta after treatment with either Eme-CD9 exosomes or CVC-labeled tau transgenic exosomes, finding that both treatments triggered a similar percentage of 5% of cells forming permeabilized galectin puncta after 72 h of treatment (Fig. 4aa). However, when treating with CVC-labeled tau transgenic exosomes, 100% of the tau biosensor cells with tau aggregation also presented galectin puncta, indicating that tau aggregation was only detected in cells with permeabilization (Fig. 4bb). There was heterogeneity in that not all tau biosensor cells with puncta double positive for rTg4510 exosomes and galectin developed tau aggregates (Fig. 4aa white middle column), accounting for 24% probably because the cells had either internalized rTg4510-brain exosomes without tau seeds or those with low levels of tau seeds that were not sufficiently high to overcome the threshold for tau aggregation [60]. Given that galectin puncta were induced by HEK293T-derived exosomes lacking tau as well as by brain-derived exosomes containing tau seeds, we conclude that the induction of endosome permeabilization is a common attribute of exosomes independent of their cargo or cellular origin.

**Fig. 5:**
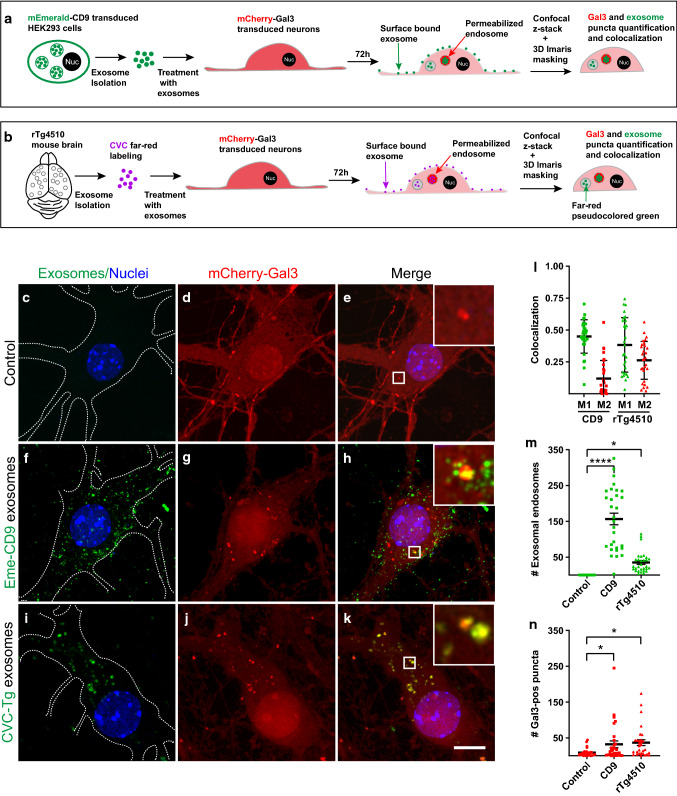
Exosomes also trigger endolysosomal permeabilization in neurons. **a**, **b** Diagrams of the experimental design. Primary neurons were transduced at DIV2 with a lentivirus for mCherry-Gal3 and treated at DIV7 with either HEK293T-derived Eme-CD9 exosomes or CVC-labeled rTg4510-brain exosomes (CVC-Tg, magenta pseudo-colored green for better visualization) and imaged with Z-stacks 72 h later (DIV10). To analyze only internalized exosomes, 3D Imaris masking was used to exclude exosomes bound to the plasma membrane. Scale bar: 10 µm for all panels. Insets show higher magnifications of the boxed areas containing puncta. **c**–**e** Control neurons showing diffuse somatic distribution of mCherry-Gal3 and the occasional puncta (magnified in inset). **f**–**h** Treatment with Eme-CD9 exosomes results in an increase in mCherry-Gal3 puncta, with evident colocalization (inset magnification). **i**–**k** CVC-Tg exosomes also induce formation of mCherry-Gal3 puncta. **l** Manders’ colocalization coefficients, with M1 representing the fraction of the exosomal signal (green) that colocalizes with mCherry-Gal3 (red). M2 represents mCherry-Gal3 signal colocalizing with the exosomal fluorophore. Error bars represent ± SD. **m**–**n** Quantification of the number of exosome-containing endosomes and galectin-3 puncta in neurons. The number of galectin-3 puncta is increased in neurons that have internalized exosomes compared to untreated controls. Error bars represent ± SEM for *n* = 30 neurons analyzed per condition. ****p < 0.0001; *p < 0.05; *ns* not significant

To investigate whether the formation of galectin puncta induced by exogenous exosomes was conserved in neurons, we next transduced mouse hippocampal neurons with a lentivirus expressing mCherry-Gal3 at DIV2, followed by treatment at DIV7 with either Eme-CD9 exosomes or CVC-labeled tau transgenic exosomes for a total of 72 h (Fig. 5a–n). We chose the 72-h time-point because our analysis of tau aggregation in neurons expressing lentiviral tau-YFP did result in tau aggregation induced by rTg4510-derived exosomes in most neurons at 72 h and was absent at 24 h (Supplementary Fig. 2), reminiscent of the above data obtained in tau biosensor cells (Fig. 4n–t) and also our previous studies [60]. As seen for HEK293T and tau biosensor cells, control neurons exhibited diffuse cytoplasmic distribution of Gal3 and minor puncta in the absence of internalized exosomes (Fig. 5c–e). However, the number of Gal3 puncta increased on average 4.0-fold with the uptake of exogenous exosomes (Fig. 5f–k, n). Internalized exosomes colocalized with Gal3 at an average of 42% (Fig. 5l), but the uptake of Eme-CD9 was ~ threefold higher (Fig. 5m), probably because CD9 is a protein involved in cell adhesion [43], which might explain why Eme-CD9 exosomes attached more strongly to neuronal plasma membranes and potentially increased exosomal uptake. Together, the galectin assay demonstrates that endosomal permeabilization occurs after endocytosis of exosomes from neurons or non-neuronal cells, constituting a gateway that could be used by exosomal tau seeds to escape from the endolysosomes.

### Damaged endolysosomes are repaired by autophagy

**Fig. 6:**
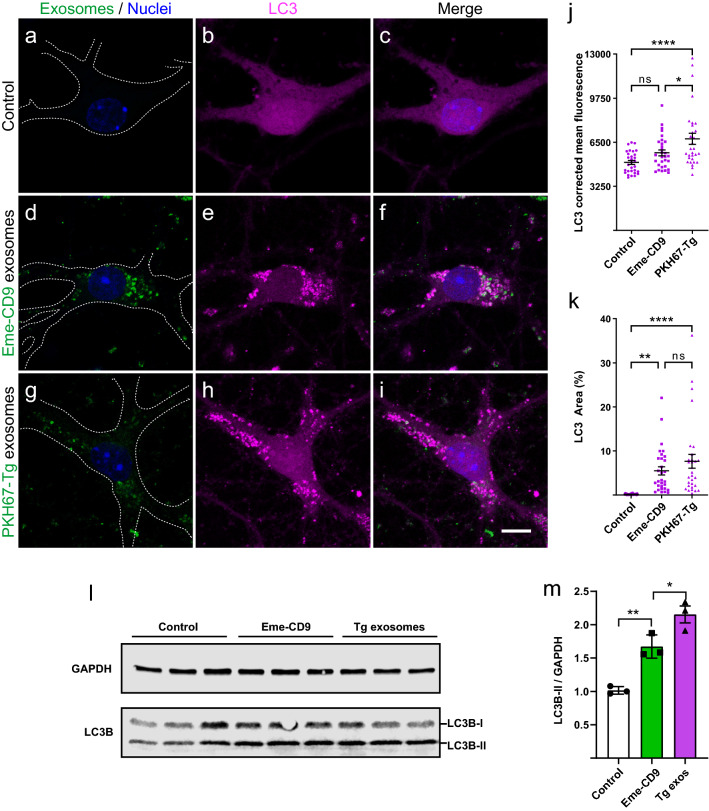
Exosome-containing endolysosomes trigger accumulation and relocalization of the autophagic marker LC3. Immunofluorescence in hippocampal neurons to detect endogenous LC3, a known marker of autophagosome formation. Neurons were treated at DIV7 with brain-derived exosomes (PKH67-Tg, green label) or HEK293T-derived exosomes (Eme-CD9) for 72 h. **a**–**c** Untreated control neurons showing a physiological distribution of endogenous LC3 (magenta). **d**–**f** Neurons treated with Eme-CD9 exosomes show strong accumulation and relocalization of endogenous LC3 at the site of exosome-containing endosomes. **g–i** PKH67-Tg exosomes also trigger the redistribution of endogenous LC3. **j**–**k** Quantification of corrected mean fluorescence for endogenous LC3 (**j**) and the percentage area of above-threshold signal (**k**) in the perinuclear region. Both metrics show a strong increase in LC3 signal upon exosome internalization. Error bars represent ± SEM for 30 individual neurons analyzed from three independent experiments. ****p < 0.0001; **p  < 0.01; *p < 0.05; ns, not significant. Scale bar: 10 µm for all panels. (**l**) Western blot analysis for LC3B in neurons treated with exosomes for 72 h. (**m**) Quantification of LC3B-II shows an increase upon treatment with exosomes, which is more pronounced with rTg4510 exosomes. Error bars represent ± SEM for three independent experiments. **p < 0.01; *p < 0.05

Permeabilization of endolysosomes results in leakage of lysosomal contents into the cytosol which potentially can lead to cell death; this explains why cells have developed mechanisms to repair or recover damaged endolysosomes [2, 37, 40, 47, 66, 75]. Autophagy-dependent recovery, which involves the engulfment of the damaged endolysosome by an autophagosome, is a mechanism by which cells avoid lysosome-dependent cytotoxicity by restoring the compartmentalization of lysosomal activity [2, 37, 40, 47, 66, 75]. Endolysosomal engulfment requires conjugation of LC3 to the expanding autophagosome membrane [37, 40, 47, 66]. Therefore, we investigated in neurons whether the autophagosomal LC3 was recruited to exosome-containing endolysosomes (Fig. 6).

Primary hippocampal neurons treated with exosomes for 72 h showed physiological expression of endogenous LC3, equally distributed throughout the cytosol of cells (Fig. 6a–c). However, neurons that had endocytosed either Eme-CD9 or rTg4510 exogenous exosomes exhibited a strong accumulation or relocalization of LC3 to perinuclear sites of exosome-containing endolysosomes (Fig. 6d–i). Furthermore, quantification of the mean fluorescence for LC3 (Fig. 6j), as well as the percentage area occupied by accumulated LC3 above a thresholded signal (Fig. 6k) in the perinuclear region, supported a strong accumulation of endogenous LC3. Neurons also showed higher levels of LC3 intensity after treatment with rTg4510 exosomes compared with Eme-CD9 exosomes, which could indicate that tau aggregates contained within rTg4510 exosomes may trigger a stronger autophagy response compared with endolysosomal permeabilization induced by Eme-CD9 exosomes which lack tau as cargo (Fig. 6j). This notion was further supported by western blot analysis showing that LC3B-II, which correlates with increased levels of autophagic vesicles [7], increased upon exosomal treatment, and even more when rTg4510 exosomes were used (Fig. 6l–m). Similarly, endogenous LC3 was also relocalized and accumulated in exosome-treated tau biosensor cells (Supplementary Fig. 3). Together, our data support the notion that autophagosomes form at the site of exosome-containing endolysosomes in a likely response to permeabilization induced by exosomal uptake (Fig. 5).

## Discussion

Tau seeds have been proposed to spread through the brain from affected to anatomically interconnected neurons, inducing tau pathology in recipient cells [15, 16, 22, 25, 27, 33, 44, 51, 58–60]. In this process, exosomes have emerged as vehicles by which neurons can secrete and pass on tau seeds [18, 58–60, 76, 77]. However, it is incompletely understood how tau-containing exosomes are propagated, how exosomal tau seeds escape the endosome, and how they induce misfolding of tau protein in the cytosol of recipient cells, converting tau from an innocuous state of a highly soluble protein into potentially neurotoxic aggregates. Here, we reveal that the induction of lysosome-mediated permeabilization of endosomes is a general cellular mechanism used by exosomes to potentially deliver cargoes that are resistant to lysosomal activity to recipient cells. In the pathological context of AD, our study supports a role for lysosomes in the escape of exosomal tau seeds from the endolysosome, allowing for the interaction between the tau seeds and endogenous cytosolic tau in recipient cells as a critical step in tau pathogenesis.

### Endosome permeabilization as a gateway to the cytosol

Exosomes are extracellular vesicles with physiological roles in cell-to-cell communication by carrying and delivering a range of bioactive molecules [49, 83]. The profound effects that exosomes exert in cells that internalize them implies that exosomes have developed efficient mechanisms to deliver their cargoes to the cytoplasm of recipient cells [49, 83]. Our study revealed that exosomes can deliver cargo molecules to the cytosol of recipient cells by triggering lysosome-mediated permeabilization of endosomes. We found that this appears to be a more general mechanism, as it operates independent of whether exosomes are brain derived or derived from non-neuronal cultured cells such as HEK293T, or even whether they carry tau or not. Endolysosomal permeabilization, however, operates not in all cells, and in our experimental system of tau biosensor cells, only around 5% displayed galectin-positive, i.e., permeabilized endosomes (Fig. 4). Interestingly, in those cells that did exhibit tau aggregation, permeabilization was detected in 100% of cells, which strongly supports the notion that endolysosomal permeabilization is the gateway by which exosomal tau seeds exit into the cytosol. Moreover, as only 5% of the cells showed permeabilization although all cells appeared to have internalized exosomes, this might signify that induction of permeabilization is a thresholded mechanism as the one operating in tau aggregation [60]. Why some cells surpass the threshold and others not is intriguing, and we speculate that high activity of enzymes controlling the integrity of endosomal membranes or high levels of intracellular signals such as calcium stored in endolysosomes could be involved [17, 40, 66]. For instance, the endosomal sorting complex required for transport (ESCRT)-III machinery is involved in repairing the membranes of damaged endolysosomes [40, 66], which indicates that endolysosomes are inherently unstable and in need of frequent repair, and might explain why a small fraction of control cells exhibited some level of physiological permeabilization (Figs. 4, 5). Interestingly, impairing the function of ESCRT proteins leads to endosomal permeabilization that promotes the escape and propagation of membrane-less tau seeds [17]. This highlights the importance of the intracellular signals regulating the ESCRT-dependent repair mechanisms of endosomal membranes in the propagation of tau seeds [40]. However, although we achieved a strong reduction in tau aggregation upon impairing lysosomal function (Figs. 3, 4), this never led to a complete elimination of tau aggregation, which signifies that escape mechanisms other than permeabilization may operate in parallel. We speculate that ‘back-fusion’ [72, 81], a mechanism by which the exosomal membrane fuses with the limiting membrane of the endosome, is a potential alternative escape mechanism for exosomal tau seeds. The question arises whether back-fusion constitutes a minor mechanism compared with endolysosomal permeabilization given that it requires a close contact between exosomal and endosomal membranes, meaning that only exosomes in the periphery of the endosomal lumen can undergo back-fusion, which likely reduces the escape of tau seeds into the cytosol.

What facilitates tau seeding in recipient cells is that exosomes have unique features that lead to prolonged endolysosomal activity and, hence, rupture. Consistent with their roles as systemic messengers that travel over long distances without being degraded, exosomes have a higher rigidity in their lipid bilayer compared to that of the plasma membrane, due to an increased content in sphingomyelin, cholesterol, and di-saturated lipids [65, 84]. Therefore, the resistance of exosomal membranes to degradation may have contributed to the relative stability of the internalized exosomes in the acidic environment of endolysosomes as evidenced by our analysis, consequently triggering a strong or prolonged endolysosomal activity. In fact, the proposed stability of exosomes in the endolysosomal compartment is not surprising, taking into account that exosomes are generated in a similarly low pH environment, ranging from pH 6.0 to 4.9 for both late endosomes and MVBs [38]. However, although exosomes appear to be very stable under acidic endolysosomal conditions, it is quite likely that not all exosomal cargoes can resist conditions of low pH and the potentially high activity of lysosomal enzymes. This implies that only biomolecules that are resistant to low pH or lysosomal degradation can escape into the cytosol by lysosome-mediated permeabilization of endosomes. Interestingly, the core of tau seeds [26, 34] has been shown to resist proteases [54, 68, 78]. We consider that the ‘core’ conformers of tau seeds could potentially resist degradation in the endolysosomal environment, which together with the lysosome-mediated permeabilization of exosomal and endosomal membranes provides a means for the escape of tau seeds from the endolysosome. More generally speaking, it may explain why the number of proteins that form aggregates in human disease is limited despite the intrinsic property of a significant fraction of cellular proteins to form aggregates [19, 20].

### Recruiting lysosomes to induce tau aggregation

To examine and quantify the delivery of exosomal tau seeds to the cytosol in recipient cells, we used tau biosensor cells [34], which are sensitive to tau seeds generated in the brains of the tau-accumulating mouse model rTg4510 in forming aggregates [60]. We demonstrated that lysosomes are important for the tau aggregation induced by exosomes by interfering with lysosomal function, as demonstrated with alkalinizing agents and by performing gain- and loss-of-function studies for RAB7. Interestingly, RAB7 has been shown to have a role in tau secretion, and the partial colocalization of tau and RAB7 in both neurons and HeLa cells could indicate that endolysosomes are involved in this process [61]. We speculate that some of the newly formed autophagosomes induced by permeabilization could enter an autophagy-mediated secretory pathway, which has been recently shown to contribute to tau secretion [42]. Thus, RAB7 and lysosomes might have a dual role by facilitating the endosomal escape of tau seeds into the cytosol, and by contributing to the spreading of tau pathology through unconventional cellular secretion of tau by secretory autophagosomes. Consistent with this, RAB7 levels are upregulated in the brains of people with mild cognitive impairment and AD, and are shown to correlate with the Braak stage, suggesting that RAB7 dysregulation and endolysosomal alterations represent early perturbations in AD [30, 31]. Together, this may signify that the management of RAB7 activity holds potential as a therapeutic target in AD [39]. Moreover, in agreement with our analysis of endogenous LC3, in both primary tauopathies and familial cases of AD, accumulation of the autophagic marker LC3 and evidence for endolysosomal leakage have been reported [57].

It has been suggested that the conformation of amyloid proteins, such as membrane-free aggregates of α-synuclein, huntingtin, and tau, dictates the potency of vesicle rupture [27]. However, Tsujimura and colleagues have also demonstrated that similar to our data, lysosomal activity is involved in triggering intracellular aggregate formation induced by α-synuclein fibrils [74]. It is tempting to speculate, as shown for α-synuclein [74] and by us here for exosomal tau seeds, that the prion-like induction of protein aggregation in other neurodegenerative diseases might also require lysosomes. We consider that both proteinaceous amyloids and exosomes share a high complexity and resistance to endolysosomal degradation, characteristics that are potentially required for the induction of a strong or prolonged lysosomal activity that could result in the transient permeabilization of the host endolysosomes.

### The endolysosomal network and pathological protein aggregation

As mentioned above, dysregulation of the endolysosomal network appears to be an early cellular phenotype in AD pathogenesis [30, 31, 52, 53, 79]. However, this dysregulation not only applies to tau-associated neurodegeneration in AD, but also appears to be a common theme in several diseases that are characterized by the aggregation of misfolded proteins [1, 28, 48]. For instance, genome-wide association studies have been instrumental in the identification of genes that are linked to an altered risk of developing neurodegenerative diseases like AD, PD, FTLD-tau, ALS and HD, with a large number of these risk genes found to be related to the endolysosomal network [1, 17, 28, 48, 50]. Taken together, this may indicate that common endolysosomal therapeutic targets can be modulated to ameliorate or prevent the pathological accumulation of diverse misfolded proteins and neurodegeneration. However, this also underscores the necessity to investigate endolysosomal dysfunction as a risk factor in neurodegenerative diseases more generally [17, 50, 52, 79]. For instance, individuals with mutations in endosomal genes, or even with decreased levels of proteins involved in maintaining the stability of endosomal membranes [17, 40, 50, 66], could present with higher endolysosomal leakage and a consequently higher risk of developing neurodegenerative diseases.

### Recovery from the permeabilization of endolysosomes

Our study illustrates the importance of permeabilization of endolysosomes in the process of tau aggregation induced by exosomes. However, endolysosomal permeabilization could be deleterious if the cells did not respond to such an insult [2, 37, 40, 47, 66, 75]. Indeed, we showed that LC3-positive autophagic structures formed at the site of exosome-containing endolysosomes (Fig. 6; Supplementary Fig. 3) as a potential response to restore cellular homeostasis by autophagy [40, 47, 66]. Furthermore, we observed that non-neuronal cells such as tau biosensor cells proliferated and did go through mitosis even whilst harboring permeabilized endosomes and tau aggregates, which indicates that cells can recover from endosomal permeabilization (Fig. 4). We speculate that the initiation of seeded tau aggregation may be restricted to a time window before autophagosomes form and surround exosome-containing endolysosomes, given that this double containment could interfere with the continuous escape of tau seeds and the ensuing aggregation process. Therefore, pharmacological stimulation of autophagy might not only contribute to the clearance of pre-existing tau pathology as previously shown [63, 64], but also may interfere with the generation of de novo tau aggregates triggered by exosomal tau seeds.

### Concluding remarks

**Fig. 7:**
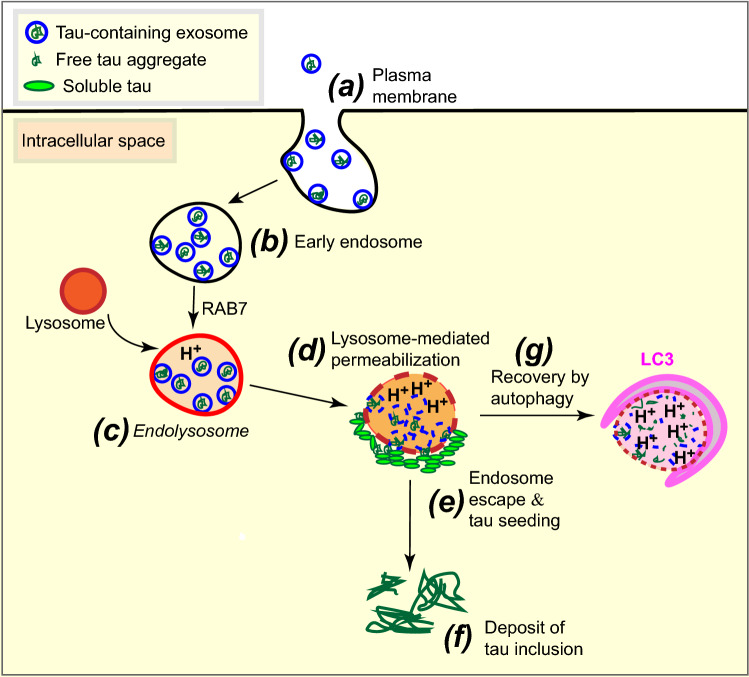
A conceptual model of the role lysosomes have in exosome-induced tau aggregation. **a** Exosomes (whether they contain tau seeds or not) enter cells by docking at the plasma membrane followed by endocytosis. **b** The internalized exosomes are contained within early endosomes. **c** RAB7-mediated tethering between endosomes and lysosomes facilitates fusion which leads to endolysosomes, with progressive increase in acidification. **d** Because lysosomal enzymes are active at low pH, exosomal degradation is being initiated but this process is lengthy as exosomes are resistant to this process (possibly because they originated in a low pH milieu), and in this process, exosomal and endosomal membranes become permeabilized. **e** Thereby, tau seeds can escape and interact with cytosolic soluble tau which they induce to aggregate. **f** As a consequence, large tau inclusions are being formed. **g** The permeabilized endolysosomes are recovered by the formation of LC3-positive autophagosomes

In conclusion, our data support our earlier studies, which demonstrated that brain-derived exosomes from tau transgenic rTg4510 mice contain corrupted forms of tau with the ability to induce tau pathology in recipient cells [5, 60]. They add to the emerging view that exosomes are more invasive than previously anticipated, acting as amplifiers in the spread of pathogenic molecules through interconnected neurons by hijacking the endosomal pathway to propagate seeds over a larger distance [59]. As summarized in our mechanistic working model for the present study (Fig. 7), exosome-containing endosomes require RAB7-mediated tethering between endosomes and lysosomes for the formation of endolysosomes as a principal cellular site of lysosomal activity [12, 38], and in the lengthy process of exosome degradation, exosomal and endosomal membranes become permeabilized granting access to the cytosol. Therefore, our work reveals a role for the permeabilization of endosomal membranes in the induction of tau aggregation induced by exosomes, and underscores the importance of the integrity of endosomal membranes in the trans-cellular invasion by aggregated proteins that are resistant to lysosomal degradation, a mechanism that appears to be shared by multiple neurodegenerative diseases.

## Supplementary Information

Below is the link to the electronic supplementary material.

Supplementary file1 (PDF 2072 KB)
