# Supplementary material for: Exosomes induce endolysosomal permeabilization as a gateway by which exosomal tau seeds escape into the cytosol
Source: Acta Neuropathol. 2021 Jan 8;141(2):235–56. doi: 10.1007/s00401-020-02254-3 (PMC7847444; doi:10.1007/s00401-020-02254-3)
Supplement: Supplementary file 1 — Supplementary file1 (PDF 2072 KB) [file 401_2020_2254_MOESM1_ESM.pdf]

Supplementary information for:

Exosomes induce endolysosomal permeabilization as a gateway by which exosomal tau seeds escape into the cytosol

Juan Carlos Polanco*, Gabriel Rhys Hand, Adam Briner, Chuanzhou Li#, and Jürgen Götz*

Clem Jones Centre for Ageing Dementia Research (CJCADR), Queensland Brain Institute (QBI), The University of Queensland, Brisbane QLD 4072, Australia.

* *Authors for correspondence:* *j.goetz@uq.edu.au* and *j.polanco@uq.edu.au*

Tel: +61-7-33466329.

\# *Current address: Department of Medical Genetics, School of Basic Medicine and Tongji Medical College, Huazhong University of Science and Technology, Wuhan 430030, China.*

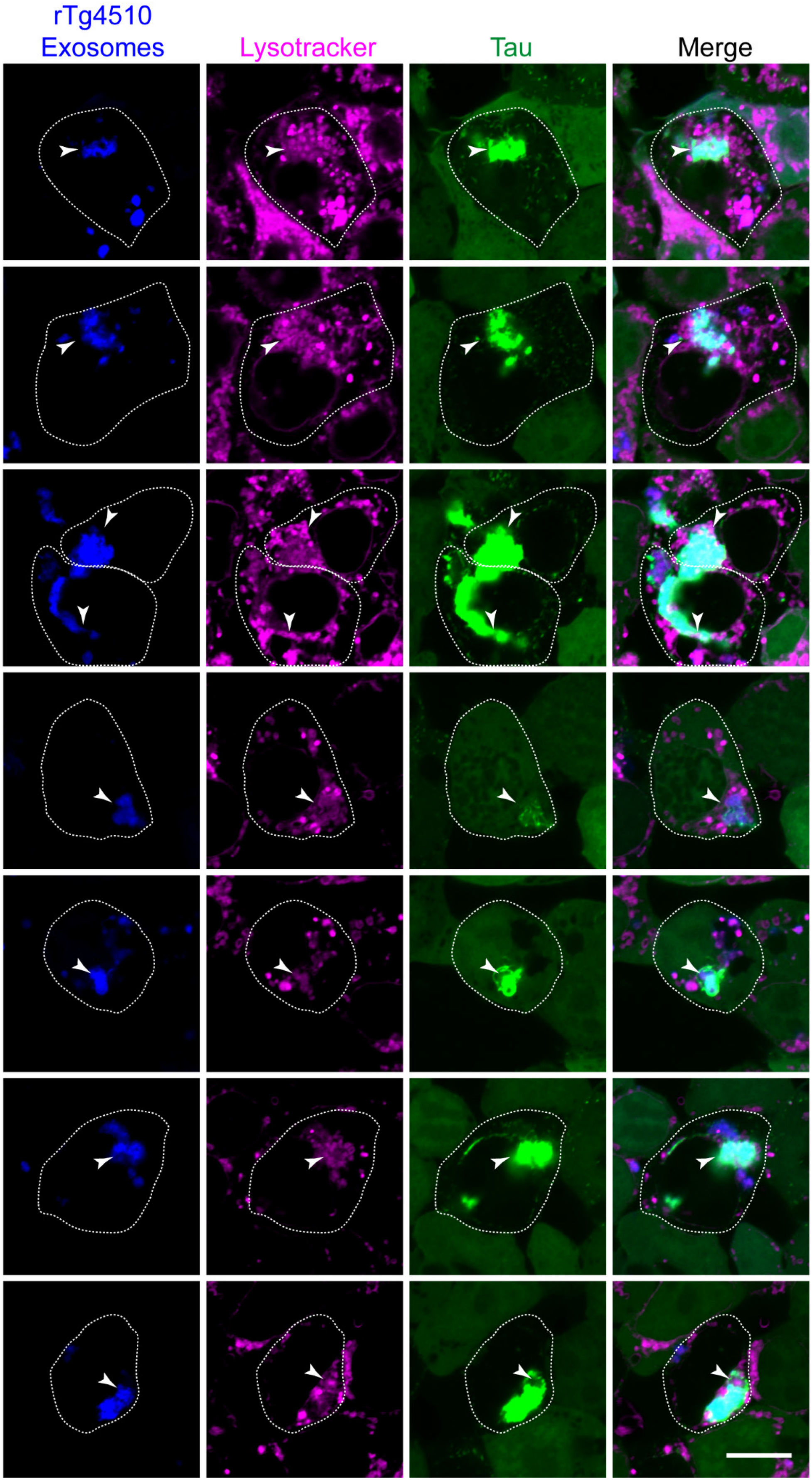

rTg4510
Exosomes
Lysotracker
Tau
Merge

Supplementary Fig 1. Additional images showing tau aggregation near the acidic Lysotracker probe colocalized with exosomes from rTg4510 samples. Lysotracker Deep Red (magenta) reveals the low pH of the endocytic organelles containing exosomes (blue, labeled with CellBrite^{TM}) in tau biosensor cells displaying tau RD-YFP in green. All the images show rTg4510-derived exosomes along with Lysotracker-positive endolysosomes, around which tau aggregates are induced (arrowheads). Individual cells are outlined with dashed lines. Scale bar: 10 µm

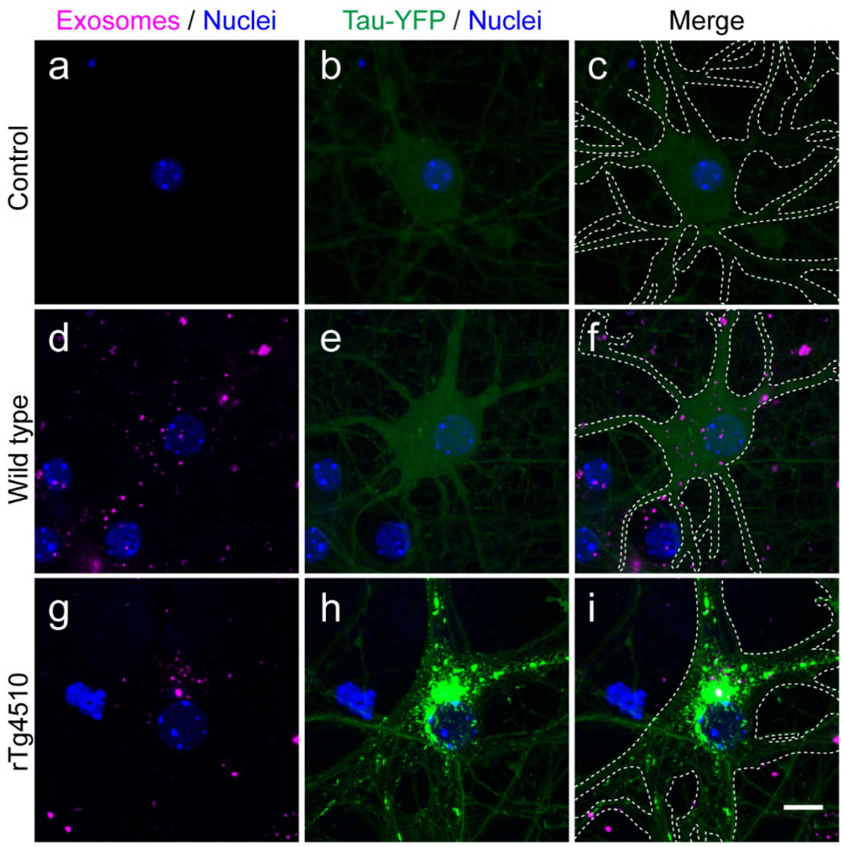

Supplementary Fig 2. Exosomes derived from tau transgenic mice induce tau aggregation in mouse neurons. Hippocampal neurons were transduced at DIV2 with a lentivirus expressing the RD domain of tau P301S fused with YFP [31]. Neurons were treated with rTg4510-brain exosomes labeled with CellVue claret Far-red dye (magenta) at DIV7 and analyzed *in vivo* every 24 h (not shown). Interestingly, aggregation was not observed at 24h, reminiscent of our previous data with tau biosensor cells revealing a time- and the concentration-dependent threshold for tau seeding by exosomes [56]. Therefore, neurons were fixed at 72h post-treatment when the highest number of complete tau inclusions were observed. Then, confocal fluorescence microscopy with Z-stacks was performed. (**a-i**) Figure shows maximum intensity projection images of Z-stacks. The dashed line outlines the plasma membrane of neurons. (**a-c**) Control neurons without exosome treatment. (**d-f**) Neurons treated with exosomes from the brains of wild-type mice. (**g-i**) Neurons treated with rTg4510-derived exosomes showing aggregates of tau RD-YFP in green. Scale bar: 10 μm for all panels.

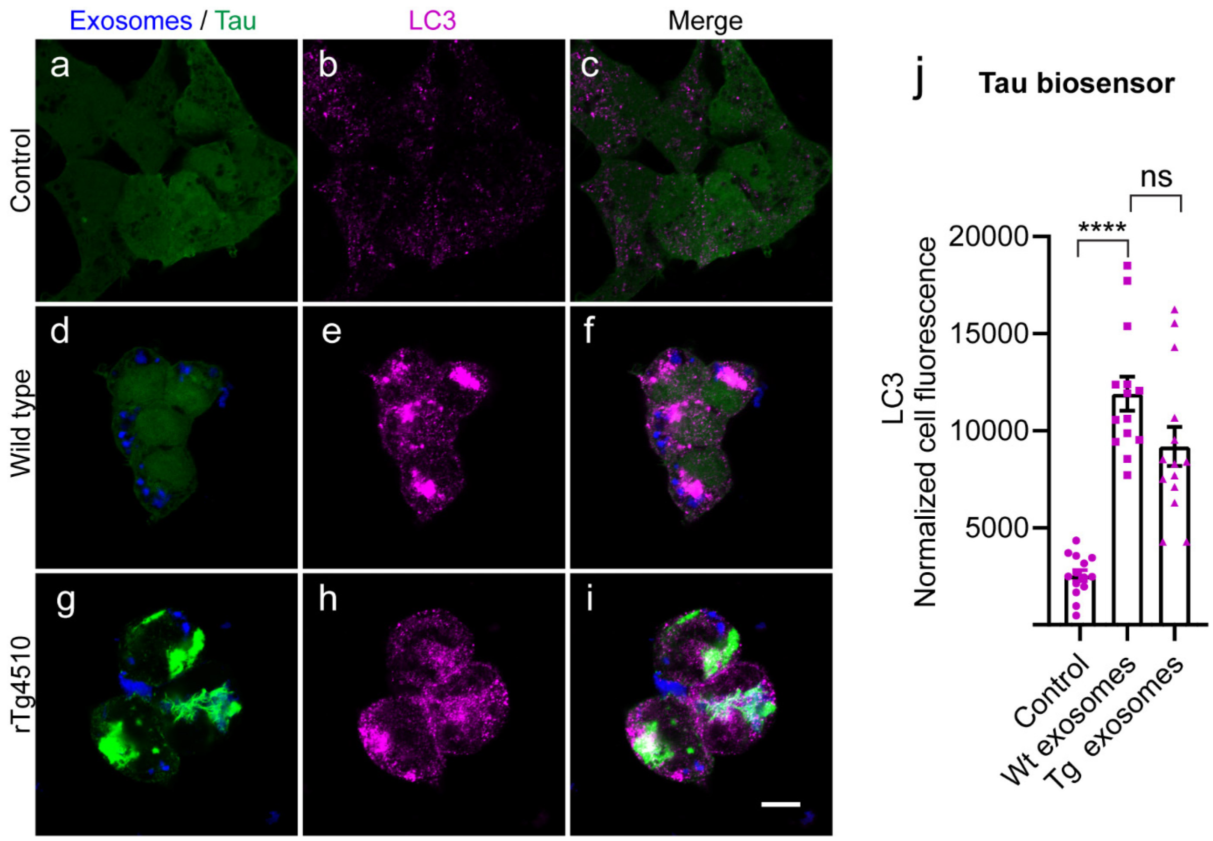

Supplementary Fig 3. Tau biosensor cells show accumulation and relocalization of the autophagic marker LC3 after treatment with exosomes. Immunofluorescence to detect endogenous LC3 in tau biosensor cells. Cells were treated with brain-derived exosomes labeled with blue CellBrite^{TM}. (**a-c**) Untreated tau biosensor cells showing tau RD-YFP in green display a physiological distribution of endogenous LC3 (magenta). (**d-f**) Tau biosensors cells treated with exosomes from wild-type brains show strong accumulation and relocalization of endogenous LC3 at the site of exosome-containing endosomes. (**g-i**) rTg4510-derived exosomes induce tau aggregation but also trigger the redistribution of endogenous LC3. Scale bar: 10 μm for all panels. (**j**) Quantification of normalized whole-cell fluorescence intensity for endogenous LC3. Error bars represent ± SEM for 15 individual cells analyzed from three independent experiments. ****$p<0.0001$; ns, not significant.
